# Death of Dr. Mower

**Published:** 1837-07-01

**Authors:** 


2. Death of Dr. Mower.

We regret to announce the death of Dr. Mower, of St. James's Place, physician to the Hospital Ship at Deptford. Dr. Mower was a very intelligent and gentlemanly man, highly respected by those who had the pleasure of his acquaintance.

